# Efficacy of Convalescent Plasma to Treat Long-Standing COVID-19 in Patients with B-Cell Depletion

**DOI:** 10.3390/life13061266

**Published:** 2023-05-27

**Authors:** Luca Tomisti, Francesca Angelotti, Mirco Lenzi, Francesco Amadori, Giovanni Sarteschi, Anna Porcu, Anna-Lisa Capria, Gloria Bertacca, Stefania Lombardi, Guido Bianchini, Antonella Vincenti, Novella Cesta

**Affiliations:** 1 ASL Toscana Nord-Ovest, Internal Medicine Department, Nuovo Ospedale Apuano, 54100 Massa, Italy; 2 ASL Toscana Nord-Ovest, Infectious Diseases Department, Nuovo Ospedale Apuano, 54100 Massa, Italy; 3 ASL Toscana Nord-Ovest, Pneumology Department, Nuovo Ospedale Apuano, 54100 Massa, Italy; 4 UOC Virologia, Dipartimento di Medicina di Laboratorio, AOUP Azienda Ospedaliero Universitaria Pisana, 56100 Pisa, Italy; 5 ASL Toscana Nord-Ovest, SSD Clinical Chemistry Analyses and Molecular Biology, Nuovo Ospedale Apuano, 54100 Massa, Italy

**Keywords:** COVID-19, COVID-19 convalescent plasma, immunocompromised patients, SARS-CoV-2, rituximab

## Abstract

The use of antivirals, corticosteroids, and IL-6 inhibitors has been recommended by the WHO to treat COVID-19. CP has also been considered for severe and critical cases. Clinical trials on CP have shown contradictory results, but an increasing number of patients, including immunocompromised ones, have shown benefits from this treatment. We reported two clinical cases of patients with prolonged COVID-19 infection and B-cell depletion who showed rapid clinical and virological recovery after the administration of CP. The first patient in this study was a 73-year-old female with a history of follicular non-Hodgkin lymphoma previously treated with bendamustine followed by maintenance therapy with rituximab. The second patient was a 68-year-old male with chronic obstructive pulmonary disease, bipolar disorder, alcoholic liver disease, and a history of mantellar non-Hodgkin lymphoma treated with rituximab and radiotherapy. After the administration of CP, both patients showed a resolution of symptoms, improvement of their clinical conditions, and a negative result of the nasopharyngeal swab test. The administration of CP might be effective in resolving symptoms and improving clinical and virological outcomes in patients with B-cell depletion and prolonged SARS-CoV2 infections.

## 1. Introduction

Since the beginning of the COVID-19 pandemic, almost 780 million people globally have been infected, and 6.9 million have died from the infection [1]. Compared to the first treatment approaches (i.e., hydroxychloroquine, azithromycin, and lopinavir), the current therapeutic scenario for SARS-CoV-2 infection has changed significantly. The new WHO guidance confirms the indication to use antivirals, corticosteroids, and IL-6 inhibitors to treat COVID-19 at different clinical stages [2]. Among the suggested therapeutic strategies, convalescent plasma (CP) has also been reported in the last WHO guidelines, although only in research settings [2] or under an Emergency Use Authorization (EUA) according to the Food and Drug Administration framework [3]. CP is a method of passive immunization, and it has been previously used as a treatment option for other viral infections, such as the Spanish influenza pandemic in 1918, the severe acute respiratory syndrome (SARS), the Middle-East respiratory syndrome (MERS), and the Ebola virus infection [4]. 

Different clinical trials on the use of CP as a treatment for SARS-CoV-2 infection have provided ambiguous results on its efficacy [5,6,7,8,9]. In fact, most of these included patients with severe forms of COVID-19, severe hypoxaemia, and the necessity for invasive mechanical ventilation. In these subjects, CP was ineffective in reducing mortality or improving the clinical condition, probably due to the extremely advanced inflammatory state of the infection [10]. 

Nevertheless, increasing clinical cases demonstrate a real benefit for patients with mild and moderate forms of COVID-19 treated with CP [11,12,13,14,15]. The major success seems to be in immunocompromised patients. In the case of treatment with rituximab, patients may develop hypogammaglobulinemia post-treatment and a failure of B-cell function recovery. Consequently, the following B-cell depletion impairs the adaptive immune response and the ability to produce neutralising antibodies; for these reasons, in B-cell-depleted subjects, the SARS-CoV-2 infection can often persist for several months [16,17,18,19,20] or rapidly evolve into the critical form [21]. In this context, using COVID-19 CP (CCP) appears promising for haematological patients, especially those who cannot produce neutralising antibodies.

We report two clinical cases of patients with B-cell depletion and prolonged COVID-19 that resulted in rapid clinical and virological recovery after the administration of CP.

## 2. Detailed Case Descriptions

**Figure 1:**
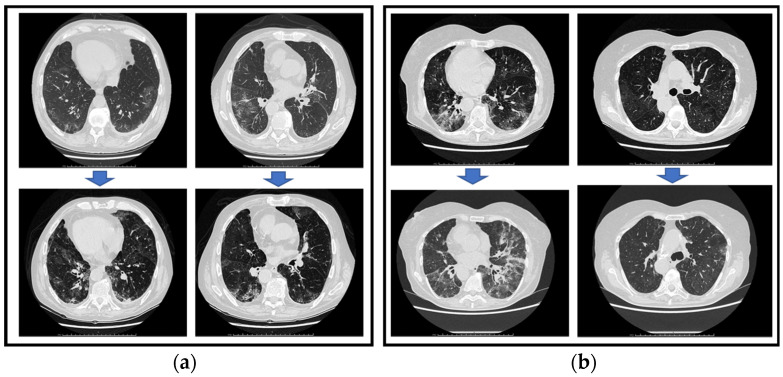
Chest CT scan before (**upper row**) and after (**lower row**) CP treatment. (**a**) Case 1; (**b**) Case 2.

Case 1: A 73-year-old female patient affected by follicular non-Hodgkin lymphoma (stage 3) since 2020. She was treated with bendamustine (until August 2020), followed by maintenance therapy with rituximab (until February 2022), obtaining remission of the disease. She was immunized with three doses of the COVID-19 mRNA vaccine. On 15 April 2022, she tested positive for SARS-CoV-2 infection with a nasopharyngeal swab (NPS). She was treated as an outpatient, and she tested negative for a new NPS after two weeks. On 10 May, she complained of a relapse of fever and dyspnea. On 11 May, she started empirical antibiotic therapy (cotrimoxazole 160 mg/800 mg q12h per os), but after a few days, she was admitted to the emergency room with type 1 respiratory insufficiency, and the SARS-CoV2 NPS confirmed a positive result. Thus, she was referred to the Department of Medicine for a relapse of the SARS-CoV2 infection. The lung CT scan showed interstitial bilateral pneumonia (Figure 1a). Remdesevir (200 mg IV, followed by 100 mg IV q24h for 4 days) was started on 10 May, together with low molecular weight heparin (LMWH), desamethasone (6 mg IV q24h), and low flow oxygen therapy, obtaining defervescence and partial improvement of clinical conditions. She was discharged on 17 May from home isolation because she was still positive for SARS-CoV-2 and on therapy with oral steroids. On June 7, her fever and dyspnea relapsed, and she was readmitted to the hospital with the NPS still positive. We measured IgM titers against the anti-spike (anti-S) and anti-nucleocapsid (anti-N) proteins of SARS-CoV-2 and found them undetectable (cut-off > 0.1 AU/mL) as well as the titer of the anti-Spike IgG (<3.8 AU/mL, cut-off >15 AU/mL). Immunophenotyping by flow cytometry confirmed a 0% presence of CD19+ B-lymphocytes. Antibiotics (piperacillin/tazobactam 4.5 gr IV q8h) and corticosteroid therapy were started on 8 June. A bone marrow biopsy was performed, and no signs of lymphoma were revealed, but there was marked hypocellularity. The patient was discharged on 26 June and was still positive (NPS). Since the patient experienced recurrent episodes of fever and cough, she was further admitted to the hospital on 10 August. A new lung CT scan showed a worsening of the bilateral interstitial infiltrations, and a blood gas analysis showed pO2 of 63 mmHg and pCO2 of 30 mmHg. The viral load on the NPS was high (Cycle Threshold (CT) 18). Whole-genome sequencing of the virus showed that the strain belonged to the BA.2 variant. On 26 August, the ethical committee of North-Western Tuscany granted permission to use COVID-19 CP, taking into account the critical clinical condition of the patient. Three CP units were administered: the first on 27 August (300 mL; IgG anti-Spike 23,469.2 Au/mL; 3332.6 BAU/mL); the second on 2 September (300 mL; IgG anti-spike 12,635.3 Au/mL; 1794.2 BAU/mL); and the third on 10 September (300 mL; IgG anti-spike >40,000 Au/mL; >5680 BAU/mL). All donors were double-vaccinated and convalescent from different SARS-CoV-2 variants. No adverse reactions during or after the CP infusion were registered. The resolution of the fever was obtained on 30 August, the dyspnea progressively improved, and the last lung CT scan (on 7 September) showed an evolution from interstitial pneumonia to fibrosis (Figure 1a). NPS became negative on 15 September, five days after the last CP administration. One month later, the patient remains asymptomatic; an additional NPS confirmed remission of the SARS-CoV-2 infection (Table 1).

Case 2: A 68-year-old male patient affected by chronic obstructive pulmonary disease (COPD) with in-home oxygen therapy, bipolar disorder, and alcoholic liver disease. In 2012, a mantellar non-Hodgkin lymphoma was diagnosed and treated with rituximab and radiotherapy, resulting in the complete remission of the disease. The patient was not vaccinated against SARS-CoV-2. On 14 July 2022, he tested positive for a NPS for SARS-CoV-2 and was admitted to the Department of Pneumology for fatigue and dyspnoea. A chest X-ray was negative for interstitial pneumonia, and the patient was treated with a 3-day course of remdesivir (200 mg IV as a loading dose, then 100 mg/day IV for 2 days, from 15 to 17 July) and then discharged. At the end of August 2022, the patient was admitted to the hospital again for high fever (39 °C) and respiratory distress; the molecular NPS resulted in a positive (CT 25). The lung CT scan showed bilateral interstitial pneumoniae (Figure 1b), and the blood exams revealed a C-reactive protein of 30 mg/dl and a d-dimer of 3300 ng/mL FEU. The dosage of anti-Spike IgM was 0.112 AU/mL (cut-off > 0.1 AU/mL), while anti-Spike IgG was <3.8 AU/mL (cut-off >15 AU/mL). Evaluation of CD19+ B-lymphocytes was 0% at the immunophenotyping by flow cytometry. The urinary antigen test was negative, as were the blood culture, urine culture, and film-array respiratory panel. A blood gas analysis revealed pO2 of 65 mmHg and pCO2 of 34 mmHg. The patient was treated with low-flow oxygen rates, steroids, and empiric antibiotic therapy (piperacillin/tazobactam 4.5 gr IV q8h and doxycycline 100 mg OS q12h, from 26 August to 6 September). Furthermore, considering the negative titre of anti-Spike IgG, the monoclonal combination tixagevimab/cilgavimab (150 mg/150 mg IM) was administered, leading to a partial reduction of fever. On 5 October, he was discharged with a molecular NPS that tested positive (Cycle Threshold 28) and steroid therapy per os (prednisolone 25 mg + 12.5 mg q24h). On 25 October, the patient returned to the hospital complaining of a high fever (40 °C) and the persistence of the SARS-CoV-2 infection. A new lung CT scan revealed a fibrotic thickening of the interstitium and bilateral ground-glass areas (Figure 1b). A bone marrow biopsy excluded a relapse of lymphoma but showed cellularity < 25%. The microbiological culture showed no evidence of bacterial infections; thus, antibiotics were stopped. The dosage of anti-Spike IgM and anti-N IgM was 0.20 AU/mL (cut-off > 0.1 AU/mL), while anti-Spike IgG was >400 AU/mL (cut-off >15 AU/mL). The administration of COVID-19 CP was considered for the patient, and on 16 November, the ethical committee of North-Western Tuscany granted permission to use it after the evaluation of the patient’s clinical history. The first COVID-19 CP units were administered on 19 November (500 mL; IgG anti-Spike >40,000 AU/mL; 5680 BAU/mL) and the second one on 24 November (500 mL; IgG anti-Spike 32,087 AU/mL; 4556 BAU/mL). Moreover, in this case, the donors were double-vaccinated and then infected by SARS-CoV2. The CP infusion was well tolerated; the day after the first CP infusion, complete resolution of the fever and clinical improvement in the patient were observed. The patient was discharged in good medical condition after the NPS became negative for SARS-CoV-2 on 26 November (Table 1).

## 3. Discussion

We described the positive clinical and virological outcomes of two patients with B-cell depletion and prolonged SARS-COV-2 infection treated with COVID-19 CP. 

The therapeutic efficacy of CP in COVID-19 has been investigated since the pandemic began when antivirals were not yet available. Unfortunately, the major clinical trials conducted have shown highly variable and discordant results, and this has prevented a clear definition of the therapeutic role of CP in SARS-CoV-2 infection [10]. Furthermore, clinical trials often do not include immunocompromised patients, such as those affected by haematological malignancies. However, international scientific societies are recognising the potential benefit of COVID-19 CP in the treatment of immunocompromised patients [22,23]. For instance, the European Conference on Infections in Leukaemia (ECIL 9) recommends high titre CP in patients with haematological and mild COVID-19 disease within 72 h of symptom onset; use is also recommended in patients with the moderate disease [22]. Likewise, the Association for Advancement of Blood and Biotherapies (AABB) recommends the use of CP in outpatients with COVID-19 and immunocompromised conditions to reduce the risk of hospitalisation from disease progression (a weak recommendation); it also agrees with the use of CP in hospitalised patients with moderate–severe disease if they are lacking anti-SARS-COV-2 antibodies [23]. 

Our presented patients had a clinical history of lymphoma treated with rituximab. The use of anti-CD20 antibodies has a profound impact on B-cell function, leading patients treated with rituximab and affected by COVID-19 to be unable to mount an antibody response [19]. Possible consequences are the risk of progression to severe disease, relapsing symptoms after an apparent clinical or microbiological remission, and prolonged virus positivity [21,24]. Thanks to the presence of neutralising antibodies (NAb), CP might be a therapeutic strategy due to its direct antiviral actions. NAbs are essential for virus clearance and protecting against viral diseases. Infusion of CP with the passive immunity driven by it can provide the NAbs that may restrain the SARS-CoV-2 infection [25].

In addition, based on the evidence collected during previous use of CP in other viral infections, CP may be efficient in the control of the over-activation of the immune system during SARS-CoV-2 infections (i.e., cytokine storm and complement activation) and in the immunomodulation of a hypercoagulable state [25].

The benefit of CP in B-cell-depleted patients has been reported in previous cases. In a patient affected by follicular lymphoma treated with rituximab and COVID-19 who required intubation and mechanical ventilation, Wright et al. reported the success of a single CP unit of 200 mL in recovering the clinical condition since the day after the administration [26]. Likewise, in a single-centre experience, Oliva et al. found survival in five out of six subjects with severe COVID-19 and cell-B lymphocyte depletion after three infusions of 300 mL of CP on alternate days [27]. A retrospective analysis of a small cohort of patients with COVID-19 admitted to the ICU described the survival of B lymphocyte-depleted patients compared to those immunocompetent after high-titre CP infusion. Thus, suggesting a potential benefit of hyperimmune plasma in patients with deficient antibody production, even with severe SARS-CoV-2 disease [28]. Furthermore, no adverse events were registered after the administration of convalescent plasma. 

A further advantage of CP in haematological patients seems to be the reduction in the duration of hospitalisation. A retrospective observation was conducted on 33 patients affected by severe COVID-19 and haematological malignancies. The results showed that patients who started plasma therapy within seven days of a SARS-CoV-2 diagnosis had a shorter hospitalisation duration than patients who received treatment later than one week. Unfortunately, there was no difference in mortality in patients receiving early versus late CP administration [29]. 

CP could be a valid therapeutic strategy to achieve clinical and microbiological recovery in prolonged COVID-19 and moderate disease, as noted in our experience. An observation in 17 patients affected by prolonged COVID-19 and without seroconversion showed that administration of CP was effective in the rapid recovery of clinical conditions (remission of fever and discontinuation of oxygen therapy) and the rapid decrease of RNAemia detected by digital droplet PCR. In addition, inflammatory markers (C-reactive protein and interleukin-6) were recorded as negative in less than one week [30]. Other studies report analogous results in immunocompromised patients with a long course of COVID-19, where the benefit has also been reported in improved lung lesions and inflammatory markers [31,32,33]. 

In Case 2, the patient received tixagevimab/cilgavimab as prophylaxis. Despite the presence of anti-Spike IgG following the monoclonal antibody injection, this was not enough to avoid the SARS-CoV-2 infection and the progression of the disease. On 26 January 2023, the Food and Drug Administration (FDA) announced the decision to suspend the authorization for tixagevimab/cilgavimab because of the lack of effect on new emergent SARS-CoV-2 variants [34]. Moreover, the other authorised anti-Spike mAbs (such as casirivimab/imdevimab and bamlanivimab/etesevimab) have become ineffective against recent Omicron sublineages and have been deauthorized by the FDA [35], leaving convalescent plasma as the only passive immunotherapy available. However, since we could not perform SARS-CoV-2 genome sequencing in the second patient, we cannot exclude the possibility that the failure of tixagevimab/cilgavimab was due to a lack of sensitivity to the virus variant that had infected the patient. 

However, while the reported clinical evidence demonstrates the potential therapeutic effect of COVID-19 CP in haematological patients, some controversies remain open, such as the correct timing of CP administration, the dosage, and the duration of treatment. 

As mentioned above, the most favourable timing for CP administration in immunocompromised patients seems to be the early phase of SARS-CoV-2 infection to provide a rapid immune response and prevent disease progression [22,23,36]. 

In one small study, the infusion of two units of COVID-19 CP resulted in a higher titre of detectable antibodies than a single administration [37]. In addition, in the case of persistent SARS-CoV-2 infection (with NPS that tested positive), as can occur in patients with B-cell function deficiency, administering two units of CP would seem more effective for viral eradication [38]. In a systematic review, Senefeld et al. similarly found that the mean number of two COVID-19 CP administrations was adopted among immunocompromised patients [17]. Further doses of CP should only be considered in the presence of virus persistence [36,38]. In fact, in our presented cases, while the second patient tested negative for NPS after two CP administrations, the first treated patient received three CP infusions due to the persistently positive test.

Currently, the use of CP is permitted as an off-label indication [31,33], and it implies authorization by the Ethics Committee. It is to be expected that this pathway can soon be simplified in order to use CP in patients who are ready candidates. In particular, we hope for the design of clinical trials that consider the use of CP in immunocompromised patients with SARS-CoV-2 infection and without seroconversion; those should also provide a better understanding of information that is now unclear, such as the optimal dose of plasma to infuse and the most effective antibody titre.

## 4. Conclusions

Our experience suggests that convalescent plasma was safe and effective in treating two B-cell-depleted patients affected by moderate and prolonged SARS-CoV-2 infections. Similar to our experience, several data points suggest that CP should be considered a therapeutic strategy in COVID-19 patients with persistent infections or without seroconversion. However, more evidence and trials are needed to establish the proper use of this therapeutic strategy.

## Figures and Tables

**Table 1:** Cycle Threshold (CT) values from qualitative PCR serial nasopharyngeal swabs (NPS).

	Before CP	After 1° CP	After 2° CP	After 3° CP	Last NPS
Patient 1	16	24	26	38	Neg
Patient 2	25	28	38	/	Neg

CP: convalescent plasma; Neg: negative.

## Data Availability

Not applicable.

## References

[1] WHO Coronavirus (COVID-19) Dashboard. https://covid19.who.int.

[2] Therapeutics and COVID-19: Living Guideline, 13 January 2023. https://www.who.int/publications-detail-redirect/WHO-2019-nCoV-therapeutics-2023.1.

[3] (2020). Investigational COVID-19 Convalescent Plasma; Guidance for Industry.

[4] Marano G., Vaglio S., Pupella S., Facco G., Catalano L., Liumbruno G.M., Grazzini G. (2016). Convalescent Plasma: New Evidence for an Old Therapeutic Tool?. Blood Transfus..

[5] Menichetti F., Popoli P., Puopolo M., Spila Alegiani S., Tiseo G., Bartoloni A., De Socio G.V., Luchi S., Blanc P., Puoti M. (2021). Effect of High-Titer Convalescent Plasma on Progression to Severe Respiratory Failure or Death in Hospitalized Patients With COVID-19 Pneumonia: A Randomized Clinical Trial. JAMA Netw. Open.

[6] Bégin P., Callum J., Jamula E., Cook R., Heddle N.M., Tinmouth A., Zeller M.P., Beaudoin-Bussières G., Amorim L., Bazin R. (2021). Convalescent Plasma for Hospitalized Patients with COVID-19: An Open-Label, Randomized Controlled Trial. Nat. Med..

[7] Heustess A.M., Allard M.A., Thompson D.K., Fasinu P.S. (2021). Clinical Management of COVID-19: A Review of Pharmacological Treatment Options. Pharmaceuticals.

[8] Li L., Zhang W., Hu Y., Tong X., Zheng S., Yang J., Kong Y., Ren L., Wei Q., Mei H. (2020). Effect of Convalescent Plasma Therapy on Time to Clinical Improvement in Patients With Severe and Life-Threatening COVID-19: A Randomized Clinical Trial. JAMA.

[9] Avendaño-Solá C., Ramos-Martínez A., Muñez-Rubio E., Ruiz-Antorán B., Malo de Molina R., Torres F., Fernández-Cruz A., Calderón-Parra J., Payares-Herrera C., Díaz de Santiago A. (2021). A Multicenter Randomized Open-Label Clinical Trial for Convalescent Plasma in Patients Hospitalized with COVID-19 Pneumonia. J. Clin. Investig..

[10] Focosi D., Franchini M., Pirofski L., Burnouf T., Paneth N., Joyner M.J., Casadevall A. (2022). COVID-19 Convalescent Plasma and Clinical Trials: Understanding Conflicting Outcomes. Clin. Microbiol. Rev..

[11] Duan K., Liu B., Li C., Zhang H., Yu T., Qu J., Zhou M., Chen L., Meng S., Hu Y. (2020). Effectiveness of Convalescent Plasma Therapy in Severe COVID-19 Patients. Proc. Natl. Acad. Sci. USA.

[12] Zhang B., Liu S., Tan T., Huang W., Dong Y., Chen L., Chen Q., Zhang L., Zhong Q., Zhang X. (2020). Treatment With Convalescent Plasma for Critically Ill Patients With Severe Acute Respiratory Syndrome Coronavirus 2 Infection. Chest.

[13] Psaltopoulou T., Sergentanis T.N., Pappa V., Politou M., Terpos E., Tsiodras S., Pavlakis G.N., Dimopoulos M.A. (2020). The Emerging Role of Convalescent Plasma in the Treatment of COVID-19. HemaSphere.

[14] Shen C., Wang Z., Zhao F., Yang Y., Li J., Yuan J., Wang F., Li D., Yang M., Xing L. (2020). Treatment of 5 Critically Ill Patients With COVID-19 With Convalescent Plasma. JAMA.

[15] Figlerowicz M., Mania A., Lubarski K., Lewandowska Z., Służewski W., Derwich K., Wachowiak J., Mazur-Melewska K. (2020). First Case of Convalescent Plasma Transfusion in a Child with COVID-19-Associated Severe Aplastic Anemia. Transfus. Apher. Sci..

[16] Thompson M.A., Henderson J.P., Shah P.K., Rubinstein S.M., Joyner M.J., Choueiri T.K., Flora D.B., Griffiths E.A., Gulati A.P., Hwang C. (2021). Association of Convalescent Plasma Therapy with Survival in Patients with Hematologic Cancers and COVID-19. JAMA Oncol..

[17] Senefeld J.W., Klassen S.A., Ford S.K., Senese K.A., Wiggins C.C., Bostrom B.C., Thompson M.A., Baker S.E., Nicholson W.T., Johnson P.W. (2021). Use of Convalescent Plasma in COVID-19 Patients with Immunosuppression. Transfusion.

[18] Focosi D., Franchini M. (2021). COVID-19 Neutralizing Antibody-Based Therapies in Humoral Immune Deficiencies: A Narrative Review. Transfus. Apher. Sci..

[19] Furlan A., Forner G., Cipriani L., Vian E., Rigoli R., Gherlinzoni F., Scotton P. (2021). COVID-19 in B Cell-Depleted Patients After Rituximab: A Diagnostic and Therapeutic Challenge. Front. Immunol..

[20] Grubovic Rastvorceva R.M., Useini S., Stevanovic M., Demiri I., Petkovic E., Franchini M., Focosi D. (2022). Efficacy and Safety of COVID-19 Convalescent Plasma in Hospitalized Patients—An Open-Label Phase II Clinical Trial. Life.

[21] Ferrari S., Caprioli C., Weber A., Rambaldi A., Lussana F. (2021). Convalescent Hyperimmune Plasma for Chemo-Immunotherapy Induced Immunodeficiency in COVID-19 Patients with Hematological Malignancies. Leuk. Lymphoma.

[22] Cesaro S., Ljungman P., Mikulska M., Hirsch H.H., von Lilienfeld-Toal M., Cordonnier C., Meylan S., Mehra V., Styczynski J., Marchesi F. (2022). Recommendations for the Management of COVID-19 in Patients with Haematological Malignancies or Haematopoietic Cell Transplantation, from the 2021 European Conference on Infections in Leukaemia (ECIL 9). Leukemia.

[23] Estcourt L.J., Cohn C.S., Pagano M.B., Iannizzi C., Kreuzberger N., Skoetz N., Allen E.S., Bloch E.M., Beaudoin G., Casadevall A. (2022). Clinical Practice Guidelines From the Association for the Advancement of Blood and Biotherapies (AABB): COVID-19 Convalescent Plasma. Ann. Intern. Med..

[24] Mehta P., Porter J.C., Chambers R.C., Isenberg D.A., Reddy V. (2020). B-Cell Depletion with Rituximab in the COVID-19 Pandemic: Where Do We Stand?. Lancet Rheumatol..

[25] Rojas M., Rodríguez Y., Monsalve D.M., Acosta-Ampudia Y., Camacho B., Gallo J.E., Rojas-Villarraga A., Ramírez-Santana C., Díaz-Coronado J.C., Manrique R. (2020). Convalescent Plasma in Covid-19: Possible Mechanisms of Action. Autoimmun. Rev..

[26] Wright Z., Bersabe A., Eden R., Bradley J., Cap A. (2021). Successful Use of COVID-19 Convalescent Plasma in a Patient Recently Treated for Follicular Lymphoma. Clin. Lymphoma Myeloma Leuk..

[27] Oliva A., Cancelli F., Brogi A., Curtolo A., Savelloni G., Siccardi G., Marcelli G., Mazzuti L., Ricci P., Turriziani O. (2022). Convalescent Plasma for Haematological Patients with SARS-CoV-2 Pneumonia and Severe Depletion of B-Cell Lymphocytes Following Anti-CD20 Therapy: A Single-Centre Experience and Review of the Literature. New Microbiol..

[28] Erber J., Wiessner J.R., Huberle C., Schneider J., Mijočević H., von Bomhard D., Luppa P., Schmid R.M., Rasch S., Lahmer T. (2021). Convalescent Plasma Therapy in B-Cell-Depleted and B-Cell Sufficient Patients with Life-Threatening COVID-19—A Case Series. Transfus. Apher. Sci..

[29] Jeyaraman P., Agrawal N., Bhargava R., Bansal D., Ahmed R., Bhurani D., Bansal S., Rastogi N., Borah P., Naithani R. (2021). Convalescent Plasma Therapy for Severe Covid-19 in Patients with Hematological Malignancies. Transfus. Apher. Sci..

[30] Hueso T., Pouderoux C., Péré H., Beaumont A.-L., Raillon L.-A., Ader F., Chatenoud L., Eshagh D., Szwebel T.-A., Martinot M. (2020). Convalescent Plasma Therapy for B-Cell–Depleted Patients with Protracted COVID-19. Blood.

[31] Belcari G., Conti A., Mazzoni A., Lanza M., Mazzetti P., Focosi D. (2022). Clinical and Virological Response to Convalescent Plasma in a Chronic Lymphocytic Leukemia Patient with COVID-19 Pneumonia. Life.

[32] Ribeiro L.C., Benites B.D., Ulaf R.G., Nunes T.A., Costa-Lima C., Addas-Carvalho M., Proenca-Modena J.L., Granja F., da Costa V.A., Duarte A.d.S.S. (2021). Rapid Clinical Recovery of a SARS-CoV-2 Infected Common Variable Immunodeficiency Patient Following the Infusion of COVID-19 Convalescent Plasma. Allergy Asthma Clin. Immunol..

[33] Ormazabal Vélez I., Induráin Bermejo J., Espinoza Pérez J., Imaz Aguayo L., Delgado Ruiz M., García-Erce J.A. (2021). Two Patients with Rituximab Associated Low Gammaglobulin Levels and Relapsed Covid-19 Infections Treated with Convalescent Plasma. Transfus. Apher. Sci..

[34] US Food and Drug Administration (2023). Announces Evusheld Is not Currently Authorized for Emergency Use in the U.S..

[35] Commissioner O. Of the Coronavirus (COVID-19) Update: FDA Limits Use of Certain Monoclonal Antibodies to Treat COVID-19 Due to the Omicron Variant. https://www.fda.gov/news-events/press-announcements/coronavirus-covid-19-update-fda-limits-use-certain-monoclonal-antibodies-treat-covid-19-due-omicron.

[36] Bloch E.M., Focosi D., Shoham S., Senefeld J., Baden L.R., Tiberghien P., Sullivan D., Cohn C., Henderson J.P., So-Osman C. (2023). Guidance on the Use of Convalescent Plasma to Treat T Immunocompromised Patients with COVID-19. Clin. Infect. Dis..

[37] Leon J., Merrill A.E., Rogers K., Kurt J., Dempewolf S., Ehlers A., Jackson J.B., Knudson C.M. (2022). SARS-CoV-2 Antibody Changes in Patients Receiving COVID-19 Convalescent Plasma from Normal and Vaccinated Donors. Transfus. Apher. Sci..

[38] Focosi D., Senefeld J.W., Joyner M.J., Sullivan D., Casadevall A., Bloch E.M., Franchini M. (2023). Lower Anti-Spike Levels in B-Cell-Depleted Patients after Convalescent Plasma Transfusion Suggest the Need for Repeated Doses. Br. J. Haematol..

